# Absence of ATG9A and synaptophysin demixing on Rab5 mutation-induced giant endosomes

**DOI:** 10.1186/s13041-024-01132-3

**Published:** 2024-09-02

**Authors:** Jiyoung Choi, Yumei Wu, Daehun Park

**Affiliations:** 1 Department of Medical and Biological Sciences, The Catholic University of Korea, Gyeonggi-Do, Bucheon, 14662 South Korea; 2 Department of Biotechnology, The Catholic University of Korea, Gyeonggi-Do, Bucheon, 14662 South Korea; 3 Departments of Neuroscience and of Cell Biology, HHMI, Program in Cellular Neuroscience, Neurodegeneration, and Repair, Yale School of Medicine, New Haven, CT 06510 USA

**Keywords:** ATG9A, Synaptophysin, Vesicle clusters, Liquid–liquid phase separation, Endosomes

## Abstract

ATG9A is the only integral membrane protein among core autophagy-related (ATG) proteins. We previously found that ATG9A does not co-assemble into synaptophysin-positive vesicles, but rather, localizes to a distinct pool of vesicles within synapsin condensates in both fibroblasts and nerve terminals. The endocytic origin of these vesicles further suggests the existence of different intracellular sorting or segregation mechanisms for ATG9A and synaptophysin in cells. However, the precise underlying mechanism remains largely unknown. In this follow-up study, we investigated the endosomal localization of these two proteins by exploiting the advantages of a Rab5 mutant that induces the formation of enlarged endosomes. Notably, ATG9A and synaptophysin intermix perfectly and do not segregate on giant endosomes, indicating that the separation of these two proteins is not solely caused by the inherent properties of the proteins, but possibly by other unknown factors.

**Supplementary Information:**

The online version contains supplementary material available at 10.1186/s13041-024-01132-3.

ATG9A, the only transmembrane protein among the core autophagy machinery, is known to localize on small vesicles [1–3]. This protein is ubiquitously expressed in most tissues and is particularly abundant in the central nervous system [4]. Recent studies have suggested the presynaptic localization of ATG9A in neurons [5–8]. In *Caenorhabditis elegans*, ATG-9 (homologs to mammalian ATG9A) vesicles are transported to presynaptic nerve terminals and undergo activity-dependent exo- and endocytosis [7]. However, the precise localization of ATG9A in nerve terminals, whether it resides on synaptic vesicles (SVs) or in distinct vesicle pools, was unclear. Using an ectopic expression system that we previously developed [9], we found that ATG9A has an ability to form vesicle condensates when it is co-expressed with synapsin [8], a peripheral SV protein that plays an important role in SV clustering [10]. The liquid-like properties of the condensates formed by ATG9A and synapsin or synaptophysin and synapsin [8, 9] suggest that the liquid–liquid phase separation (LLPS) principle could account for the clustering of two different types of vesicles. Notably, when both ATG9A and synaptophysin were overexpressed along with synapsin, the ATG9A and synaptophysin vesicle condensates were juxtaposed but clearly segregated [8] (Fig. 1A). Correlative light and electron microscopy (CLEM) further revealed a size difference between the two types of vesicles; ATG9A-positive vesicles were slightly larger than synaptophysin-positive vesicles as previously reported [8] (Fig. 1B).

**Fig. 1:**
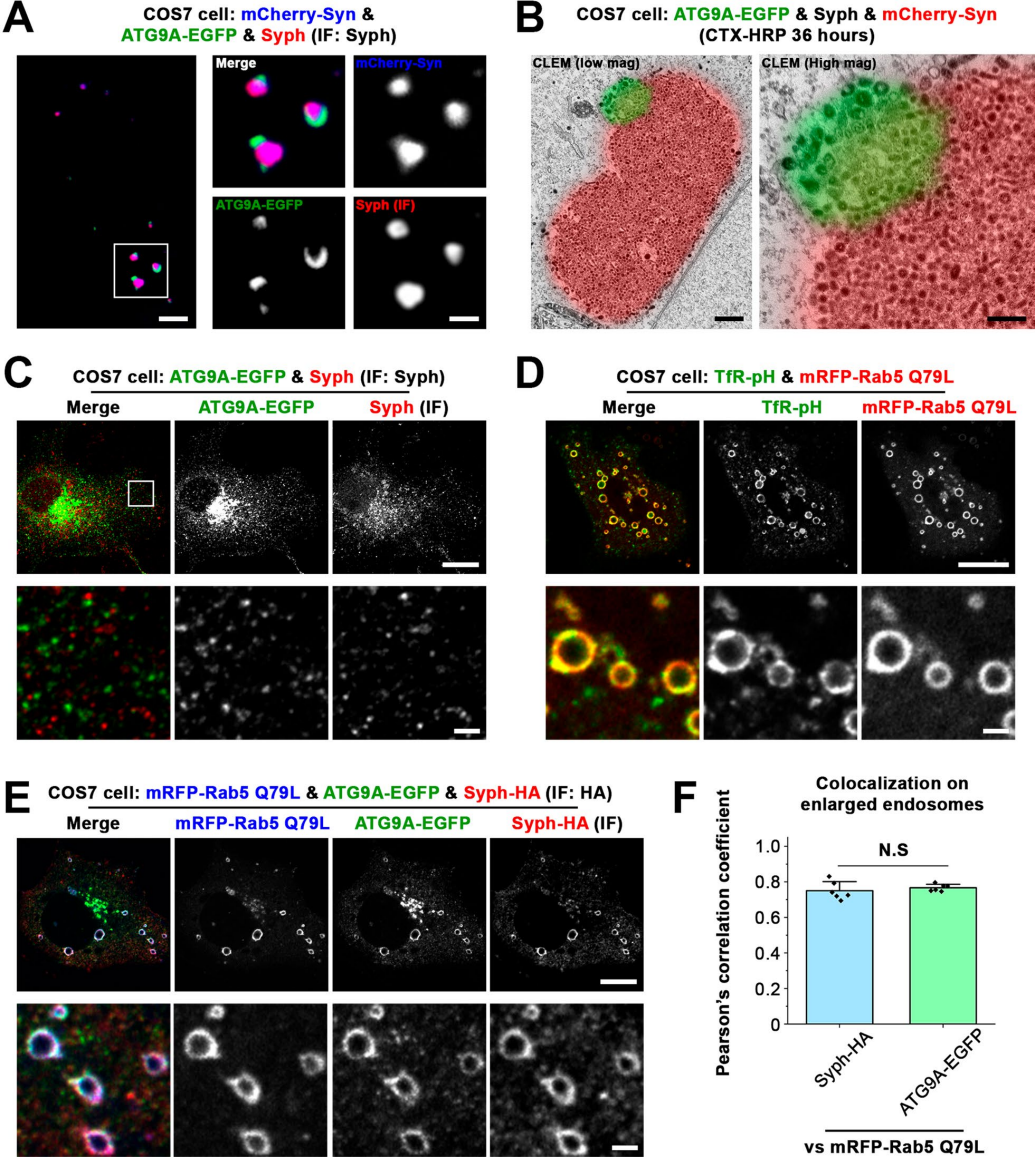
Absence of synaptophysin and ATG9A segregation on Rab5 mutant giant endosomes. **A** COS7 cells were triple co-transfected with ATG9A-EGFP, synaptophysin (untagged), and mCherry-synapsin. Synaptophysin was detected by immunofluorescence (IF). **B** COS7 cells co-expressing ATG9A-EGFP, synaptophysin (untagged), and mCherry-synapsin were incubated with 10 μg/ml cholera toxin-conjugated HRP (CHX-HRP) for 36 h and then fixed for correlative light and electron microscopy (CLEM). **C** Co-expression of ATG9A-EGFP and synaptophysin (untagged) in COS7 cells. Synaptophysin was visualized by IF. **D** Co-expression of transferrin receptor-pHluorin (TfR-pH) and mRFP-Rab5 Q79L in COS7 cells. **E** COS7 cells were triple transfected with mRFP-Rab5 Q79L, ATG9A-EGFP, and synaptophysin-HA. Synaptophysin-HA was detected by IF using anti-HA antibodies. **F** The colocalization of mRFP-Rab5 Q79L with synaptophysin-HA or with ATG9A-EGFP on the enlarged endosomes was analyzed using Pearson’s coefficients. Values are presented as means ± SD; N.S., not significant by Student’s *t*-test, n = 6 different cells for each group. Scale bars, **A** 5 μm (left) or 2 μm (right). **B** 500 nm (left) or 200 nm (right). **C**–**E** 20 μm (top) or 2 μm (bottom)

Here, we additionally found that, in the absence of synapsin, both ATG9A-EGFP- and synaptophysin-containing vesicles failed to form condensates, but these vesicles showed a scattered punctate expression pattern throughout the cytoplasm with very low colocalization (Fig. 1C). These results clearly indicate that synaptophysin and ATG9A localize to distinct vesicle pools, regardless of synapsin expression. Thus, synapsin may simply enhance the clustering of vesicles, while ATG9A and synaptophysin possibly have intrinsic demixing properties or different sorting mechanisms. Both ATG9A- and synaptophysin-positive vesicles were labeled by the endocytic tracer, cholera toxin-horseradish peroxidase (CTX-HRP) (Fig. 1B), suggesting that both ATG9A- and synaptophysin-positive vesicles undergo exo- and endocytosis (Fig. 1B).

Thus, an intriguing question arises regarding when and where these two proteins are sorted differently. Since a fraction of synaptophysin or ATG9A is believed to localize on endosomes [1, 2, 11, 12], which are key intracellular sorting organelles especially after certain proteins are endocytosed, we speculated that synaptophysin and ATG9A might form microdomains or condensates on the endosomes to facilitate their distinct sorting. We therefore tested their potential segregation on endosomes by utilizing a GTPase-defective mutant form of Rab5 Q79L, which is known to form enlarged early endosomes [13]. As previously described [13], expression of the mRFP-conjugated Rab5 Q79L mutant in fibroblasts induced the formation of large endosomes positive for transferrin receptors that undergo endosomal recycling (Fig. 1D). When Rab5 Q79L was co-expressed with ATG9A-EGFP and synaptophysin-HA, the two proteins were localized on the same mRFP-Rab5 Q79L-positive giant endosomes (Fig. 1E), however, unlike their distinct localization with or without synapsin (Fig. 1A–C), both proteins were well-mixed on the same endosome (Fig. 1E, F). These results indicate that ATG9A and synaptophysin lost their ability to segregate, at least on the endosome.

Thus, synaptophysin and ATG9A themselves may not have an intrinsic property that provides any repulsive force for segregation, suggesting that the demixing of two vesicles within synapsin condensates could be caused by other unrevealed factors such as vesicle sizes, lipid compositions, and/or unidentified interactors that need to be addressed in the future.

Furthermore, it is crucial to investigate the colocalization of ATG9A and synaptophysin on normal endosomes in future studies, considering that we utilized a mutant form of Rab5 to produce enlarged endosomes. While this method enhances resolution, protein overexpression can introduce artifacts [14]. Notably, Rab5 mutant-induced enlarged endosomes exhibit mixed characteristics of both early and late endosomes [14]. Advanced imaging techniques, such as super-resolution microscopy, can be used to visualize the precise endosomal localization of both proteins at endogenous levels.

The most intriguing question is the sorting mechanisms of the two proteins in nerve terminals. Our previous study and recent proteomic analysis clearly revealed that ATG9A vesicles are not SVs but represent unique vesicle pools in nerve terminals [5, 8]. Along the axons of cultured neurons, ATG9A and synaptophysin immunoreactivities were juxtaposed each other but were not perfectly colocalized [8] (Supplementary Fig. 1). A recent finding further showed that ATG9A vesicles isolated from rat brains generally lack proteins involved in budding, targeting, docking, and fusion, which are abundant in SVs [5]. Thus, it is plausible that ATG9A has different sorting mechanisms at the presynapses, which may be evolutionarily conserved among different species or cell types [8]. Finally, understanding the distinct trafficking, sorting, and biogenesis of ATG9A vesicles at presynapses can provide insights into the functioning of these vesicles in neurons and how they can be incorporated into presynaptic autophagy and related diseases.

## Methods

### Plasmid DNA construction

The following plasmids were previously described: ATG9A-EGFP [8], synaptophysin (untagged) [9], synaptophysin-HA [8], mCherry-synapsin [9], and Transferrin receptor-pHluorin (TfR-pH) [8]. mRFP-Rab5 Q79L was generated using site-directed mutagenesis (QuikChange II XL, Agilent Technologies, Santa Clara, CA, USA) of mRFP-Rab5 [15].

### Antibodies

The following primary and secondary antibodies were used: anti-synaptophysin (101 002, Synaptic Systems GmbH, Goettingen, Germany), anti-HA (MMS-101R, BioLegend, San Diego, CA, USA), Alexa Fluor 594 anti-rabbit IgG (A11037, Invitrogen, Carlsbad, CA, USA), and Alexa Fluor 594 anti-mouse IgG (A21203, Invitrogen).

### Cell culture and transfection

COS7 cells were grown in Dulbecco’s Modified Eagle’s Medium (DMEM) supplemented with 10% FBS and P/S (100 U/ml penicillin and 100 mg/ml streptomycin). Cells were maintained at 37 °C in a 5% CO_2_ humidified incubator and were transfected as previously described [8, 9].

### Correlative light and electron microscopy (CLEM) and fluorescence imaging

COS7 cells were prepared, incubated with CTX-HRP, fixed, and imaged using confocal and electron microscopes as previously described [8, 9].

### Immunofluorescence and fluorescence imaging

COS7 cells were fixed with 4% PFA in phosphate buffer (PB) containing 4% sucrose for 15 min at room temperature (RT) and washed with phosphate-buffered saline (PBS). Thereafter, cells were blocked with 3% bovine serum albumin (BSA) and 0.2% Triton X-100 in PBS for 30 min at RT. Primary and Alexa Fluor-conjugated secondary antibodies were then incubated in this buffer. Cells were incubated with primary antibodies for 1 h at RT, washed with PBS, incubated with Alexa Fluor-conjugated secondary antibodies for 45 min at RT, and washed again. Samples were mounted in Prolong Gold (Invitrogen) and imaged with a spinning disk confocal microscope (planar Apo objective 60×, 1.49-NA) and EM-CCD camera (C9100‐50; Hamamatsu Photonics, Shizuoka, Japan), controlled by the Improvision UltraVIEW VoX system (PerkinElmer, Waltham, MA, USA).

### Statistical analysis

Student’s two-sample t-tests were used to compare two independent groups. All representative fluorescence images were obtained from at least three independent experiments with similar results. Images were analyzed using the ImageJ 1.53q (National Institute of Health, Bethesda, MD, USA). Sigma Plot version 10.0 (Systat Software Inc., San Jose, CA, USA), Origin 2022b (OriginLab Corporation, Northampton, MA, USA) and SPSS (IBM Corp., Armonk, NY, USA) were used for statistical analyses.

### Supplementary Information

Supplementary Material 1.

## Data Availability

All data supporting the findings are included and are available on request from the corresponding author.

## Abbreviations

ATG9A: Autophagy related 9A
CLEM: Correlative light and electron microscopy
CTX-HRP: Cholera toxin-horseradish peroxidase
IF: Immunofluorescence
LLPS: Liquid–liquid phase separation
SV: Synaptic vesicle
Syn: Synapsin
Syph: Synaptophysin
TfR: Transferrin receptor
