# Supplementary material for: Absence of ATG9A and synaptophysin demixing on Rab5 mutation-induced giant endosomes
Source: Mol Brain. 2024 Sep 2;17:63. doi: 10.1186/s13041-024-01132-3 (PMC11367939; doi:10.1186/s13041-024-01132-3)
Supplement: Supplementary file 1 — Supplementary Material 1. [file 13041_2024_1132_MOESM1_ESM.pdf]

Supplementary Information

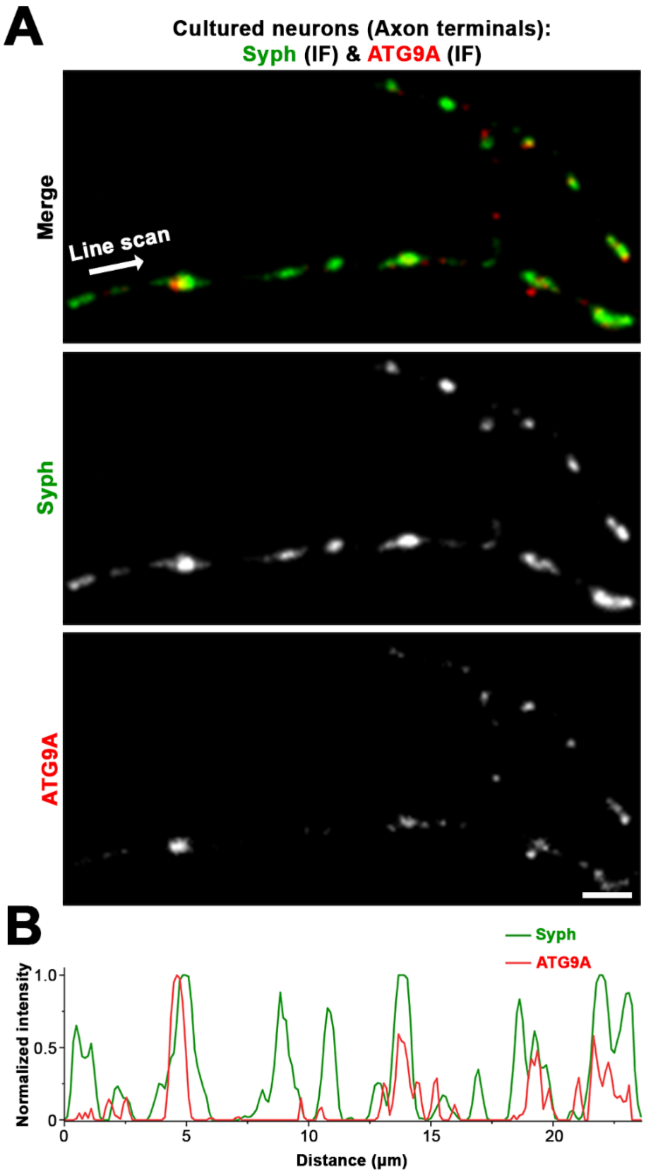

Supplementary Figure 1. Localization of ATG9A and synaptophysin in axon terminals. A, B Mouse hippocampal neuronal cultures were fixed and stained with synaptophysin (green) and ATG9A (red) antibodies at DIV17. **A** Representative confocal images. **B** Corresponding line-scan analysis of the axon from the neuron shown in A.
